# Forme atypique d'une maladie de Lyme

**DOI:** 10.11604/pamj.2015.21.100.6354

**Published:** 2015-06-09

**Authors:** Madiha Mahfoudhi, Sami Turki, Adel Kheder

**Affiliations:** 1 Service de Medecine Interne A, Hôpital Charles Nicolle, Tunis, Tunisie

**Keywords:** Dermatomyosite, maladie de Lyme, antibiotiques, Dermatomyositis, Lyme disease, antibiotics

## Abstract

La maladie de Lyme est une zoonose qui se manifeste essentiellement par des signes cutanés, articulaires, neurologiques et cardiaques. Elle peut exceptionnellement mimer une dermatomyosite. Nous rapportons l'observation d'une patiente âgée de 37 ans qui a présenté des myalgies et un érythroedème de la face et en péri-orbitaire. Une dermatomyosite a été fortement suspectée devant une élévation des enzymes musculaires. Elle a été traitée par une corticothérapie à forte dose. L’évolution a été marquée par l'aggravation des myalgies et l'apparition d'un déficit musculaire. Elle a été alors hospitalisée dans notre service. Les enzymes musculaires étaient élevées. L’électromyogramme était sans anomalies. Le bilan immunologique était négatif. Une enquête infectieuse a été réalisée retrouvant une sérologie de Lyme positive. Après administration d'une antibiothérapie adaptée, l’évolution était bonne avec disparition du tableau clinico-biologique de dermatomyosite. La précocité du diagnostic et l'instauration d'un traitement efficace permet d’éviter des complications graves.

## Introduction

La borréliose de Lyme est une zoonose d'origine bactérienne transmise par les tiques qui se manifeste le plus fréquemment par des atteintes cutanées, articulaires, neurologiques et cardiaques. De rares cas de myosites peuvent compliquer ou révéler une maladie de Lyme, responsables d'un retard diagnostique et thérapeutique.

## Patient et observation

Il s'agit d'une patiente âgée de 37 ans qui a présenté des myalgies diffuses et un érythroedème de la face et en périorbitaire. Une dermatomyosite a été fortement suspectée surtout devant une discrète élévation des enzymes musculaires. Elle a alors été traitée par un médecin de libre pratique par une forte dose de corticothérapie. L’évolution a été marquée par l'aggravation des myalgies et l'apparition d'un déficit musculaire. Elle a été alors hospitalisée dans notre service. A l'examen, elle était apyrétique, elle avait un érythroedème de la face, un déficit de la ceinture pelvienne sans déficit périphérique. L'examen neurologique était normal. A la biologie, elle avait un syndrome inflammatoire et une élévation des enzymes musculaires (CPK, LDH, Aldolase) à une fois et demi la normale. L’électromyogramme était sans anomalies. Les AAN notamment les anti JO1 étaient négatifs. L’échographie cardiaque était normale. Devant l'aggravation sous corticoïdes et la négativité du bilan immunologique, une origine infectieuse a été recherchée. La sérologie de Lyme était positive dans le sang et négative dans le LCR. La biopsie musculaire a été refusée par la patiente. Tout le reste du bilan infectieux était négatif. Sous antibiothérapie (doxycycline: 100 mg x 2/ j), l’évolution était marquée par une amélioration de son état général, disparition des myalgies et normalisation des enzymes musculaires. Notre patiente a présenté une forme atypique puisque les manifestations cliniques étaient limitées à une asthénie et des myalgies. Ces signes isolés ont rendu le diagnostic difficile.

## Discussion

La maladie de Lyme est une borréliose transmise par les tiques qui se manifeste le plus fréquemment par des atteintes cutanées (érythème migrant), articulaires, neurologiques et cardiaques [1, 2]. De rares cas de fibromyalgies [1], de myosites [2] et plus rarement de dermatomyosites [3–5] ont été publiées parmi les manifestations de la maladie de Lyme. Reimers et al. [2] ont publié une série de 8 patients atteints d'une maladie de Lyme compliquée d'une myosite avec une discrète élévation des enzymes musculaires. Ils ont rapporté la présence d'ADN borrélien dans 6 cas et l’évolution favorable sous seule antibiothérapie dans tous les cas. La recherche d'ADN sur une biopsie musculaire peut être négative mais une biopsie négative n’élimine pas la présence du spirochète à un autre endroit dans le muscle [5]. Dans certaines observations, le tableau de dermatomyosite est bruyant avec une forte élévation des enzymes musculaires. Une corticothérapie associée à l'antibiothérapie est nécessaire pour obtenir la guérison [4]. Un diagnostic précoce de la maladie de Lyme, qui parait difficile dans les formes trompeuses tel que le cas de notre patiente, est le seul garant d'un traitement efficace permettant d’éviter des complications graves cardiaques et neurologiques.

## Conclusion

La dermatomyosite constitue exceptionnellement un diagnostic différentiel de la borréliose de Lyme. La précocité du diagnostic et du traitement permet d'améliorer l’évolution.
